# Neurocognition in Congenital Central Hypoventilation Syndrome: influence of genotype and ventilation method

**DOI:** 10.1186/s13023-020-01601-7

**Published:** 2020-11-17

**Authors:** Ha Trang, Pauline Bourgeois, Fawzia Cheliout-Heraut

**Affiliations:** 1 Centre de référence de Maladies Respiratoires Rares Syndrome d’Ondine, Hôpital Universitaire Robert Debré, 48 Boulevard Sérurier, 75019 Paris, France; 2 Université de Paris, 10 avenue de Verdun, 75010 Paris, France

**Keywords:** Autonomous nervous system, Congenital Central Hypoventilation Syndrome, Neurocognition, PHOX-2B, Processing speed, Working memory

## Abstract

**Background:**

Congenital Central Hypoventilation Syndrome (CCHS) is characterized by central hypoventilation due to abnormal autonomic control of breathing and global dysautonomia. Patients harbour heterozygous *PHOX-2B* gene mutations which are polyalanine repeats of various lengths in most of the cases. A few previous studies have reported learning difficulties and neuropsychological disorders in patients with CCHS. The aims of the present study were (1) to explore the intellectual abilities of a group of children with CCHS followed up in the centre of reference for CCHS in France using the Wechsler batteries of tests, (2) and to assess whether there was any association between CCHS characteristics and various domains of the intellectual functioning.

**Results:**

There were 34 consecutive patients (15 males, 19 females) of mean (SD) age of 7.8 (3.8) years, ranging from 4 to 16 years and 6 months. Mean score of full-scale intelligence quotient was 82 (20), being in the low average range. Indexes of working memory and processing speed were significantly lower as compared to the other Wechsler indexes. There were two important findings: (1) full-scale intelligence quotient as well as indexes of verbal comprehension and processing speed were significantly greater in patients with mask ventilation than in those with tracheostomy ventilation (*p* = 0.012, 0.032 and 0.042 respectively); (2) most interestingly, in the patients with polyalanine repeats mutations, all intellectual indexes negatively correlated with the number of polyalanine expansion, with statistical significance reached for indexes of fluid reasoning and working memory (*R* = − 0.449, *p* = 0.032 and *R* = − 0.562, *p* = 0.012 respectively).

**Conclusions:**

CCHS increased the risk to develop neurocognitive deficiencies, affecting particularly speed of processing and working memory. Our results suggested that both genetics and ventilation method could be also involved in the physiopathology of neurocognitive impairment. Further investigations were required to untangle the complex underlying processes. Neurocognitive assessments should be performed regularly in children with CCHS in order to plan re-education programs, adapt school integration and improve quality of life.

## Introduction

Congenital Central Hypoventilation Syndrome (CCHS) is a rare condition of central hypoventilation due to abnormal autonomic control of breathing and global dysautonomia [15, 28]. The incidence has been estimated to be at 1/148,000–1/200,000 live births and the prevalence at 1/500,000 individuals [23, 26]. CCHS manifests at birth in most of the cases, but may also present later during childhood and even adulthood. Patients exhibit severe alveolar hypoventilation and central apnoeas, mainly during sleep, due to abnormally reduced or absent ventilatory responses to hypercapnia and hypoxia. CCHS may be isolated or associated with other dysautonomia-related conditions such as Hirschsprung disease or neural crest tumours [28]. Mutations of the paired like homeobox 2b gene (*PHOX-2B*) are found in most patients. The most frequent mutation is a heterozygous duplication of tracts of different lengths of the polyalanine stretch in the exon 3 (polyalanine repeat mutations, PARM). The number of repeats is normally set at 20 repeats, but may be expanded to 24 to 33 repeats on the affected allele in patients with CCHS [1, 21]. The length of PA expansion has been suggested to be associated with severity of autonomic dysfunction and severity of respiratory deficiency in CCHS [13]. Less common are non-PARMs (NPARMs) including missense, nonsense and frameshift mutations. Lifetime assisted ventilation and close multidisciplinary care allow the patients to have a good quality of life [9].

Neurodevelopmental and cognitive outcomes are of increased concern in CCHS [4, 9, 14, 16, 20, 22, 24, 29, 30]. However, only a few data are available. Most studies showed a high variability in the developmental and adaptive functions of the patients in their daily life. Patients had significant difficulties in the mental functions of control over motor and emotional events at the body level. Motor and speech delays were frequently reported. Also, the burden of social interactions with health services or educators along with the disease course often resulted in uncomfortable situations and was thought to limit the patients’ personal achievement [16]. Although most children attended regular schools, many had learning difficulties resulting in poor academic achievement.

A few studies showed that patients with CCHS had a wide range in intellectual indices with the Full-scale IQ being in the normal or low average range [9, 20, 30]. Attention and concentration disorders were highlighted, as well as visual/auditory memory disorders. A variety of domains were affected such as language, spatial perception, memory and executive functioning. A slow cognitive processing has been identified in one-third of the patients in an early study [24]. Mental and motor development were found normal in preschool children with *PHOX2B* 20/25 genotype and delayed in those with 20/26 and 20/27 [4]. However, these differences have not been confirmed by the same authors when they investigated school-age children with CCHS of their cohort [30]. Because recurrent hypoxia and hypercapnia are known factors that may negatively impacted brain development in the first years of life, this has lead many authors to recommend close monitoring of respiration and ensuring the best quality of oxygenation and ventilation in the patients.

The aims of the present study were to explore the intellectual abilities of a group of patients with neonatal and childhood CCHS followed up in the Centre of reference for CCHS in France and to assess whether there was any association between CCHS characteristics and various domains of the intellectual functioning.

## Methods

### Participants

The study group is composed of 34 consecutive patients with CCHS, aged from 4 to 16 years and 6 months at the time they underwent neurocognitive testing between March 2018 and October 2019. Patients originated from various parts of France and were admitted for clinical assessments in the national Center of reference for CCHS, University Hospital Robert Debré, Paris. Socio-demographic data, genetic data and medical history were reviewed. In addition to the CCHS-related assessments, neurocognitive testing was performed as recommended in routine care of all patients with CCHS in order to diagnose developmental and learning disorders. All parents gave their informed consent to publication of results in an aggregated form.

### Procedure and instruments

The patients were assessed by a licensed clinical neuropsychologist. All the testing was performed in the same period of the day, i.e. early afternoon. All patients were in good health; they spontaneously breathed room air during the procedure and wore corrective glasses or lenses if needed. The Wechsler battery of tests was used to assess intelligence efficiency and general cognitive capacities: the Wechsler for Preschool and Primary Scale for Intelligence test version IV (WPPSI-IV) was used for children younger than 6 years and the Wechsler Intelligence Scale for Children test version V (WISC-V) for those who were older. The battery of tests consisted of subtests that aim to assess specific cognitive abilities. Subtest scores scaled from 1 to 19 (knowing that the general population had a mean score of 10 and a standard deviation (SD) of 3). They were summed and converted to the Full-scale IQ (FSIQ) that represented the child’s general intellectual ability, as well as to five main primary index scores, each of them representing the abilities in discrete cognitive domains. In brief, Verbal Comprehension Index (VCI) mainly measured the ability to understand and retain verbal information (crystallized intelligence), to express and categorize; Visuo-Spatial Index (VSI) measured the ability to understand spatial relationships; Fluid Reasoning Index (FRI) measured the ability to reason and create new links (fluid intelligence); Working Memory Index (WMI) measured the ability to retain and process new information, and Processing Speed Index (PSI) measured speed of information processing. Each of these scores was set to have a mean of 100 and a SD of 15 for the general population.

### Statistical analyses

SPSS version 22 (IBM SPSS, NY, USA) was used for analyses. Data were given as means and SD for quantitative variables and absolute values for qualitative variables. The hypothesis of normality was tested using Kolmogorov–Smirnov and Shapiro–Wilk tests. Differences in continuous variables between groups were tested using the Student’s *t* test for paired samples or one way ANOVA with Tukey’s HSD post hoc tests where appropriate. In addition, as the length of polyalanine expansion is thought to be associated with the severity of autonomic dysautonomia [13], we assessed the relationship between polyalanine expansion length and intellectual indexes using Pearson’s correlations in the PARM subgroup (n = 27). All tests were two-tailed and conducted at a 5% significance level.

## Results

*The study included* 34 consecutive patients of mean (SD) age of 7.8 (3.8) years, ranging from 4 to 16 years and 6 months. Table 1 summarizes patients’ socio-demographic, genetic and clinical characteristics. The distribution of males and females was equivalent (15 and 19 respectively) as well as the distribution of children younger than 6 years and those older (16 and 18 respectively). All patients had *PHOX-2B* mutations: 27 had PARMs [(mean length of polyalanine expansion was 6.2 (1.5)] and 7 had NPARMs. All had neonatal onset of CCHS except two in whom diagnosis was made at 18 months and 9 years of age. CCHS was isolated in 27 patients and associated to Hirschsprung disease in the others. All patients received night ventilation except one. The distribution of mask ventilation and tracheostomy ventilation was equivalent (16 and 17 respectively). Of note, 14 patients received mask ventilation since the time of diagnosis and were never tracheotomised. None required 24/24 h ventilation or respiratory or cardiac pacemaker. One patient has been diagnosed with autism spectrum disorder. All lived at home with their families. Patients with CCHS had a poor academic record: although 30 of them attended regular school, 8 had repeated a school grade and 10 benefited the presence of a special needs assistant, whereas the remaining 4 required special education outside the regular system.

**Table 1:** Data of the whole group of 34 CCHS patients, including genetic and clinical data (panel A) and intellectual indices (panel B)

Panel A	
**Socio-demographic data**	34 patients
Age, mean ± SD, range, year:month	7.8 ± 3.8, 4:0–16:6
Younger than 6 years versus older than 6 years, n	16 versus 18
Male versus female, n	15 versus 19
**Genetic data**	
Patients with PARMs, n, genotype	27 [n = 8 (20/25), n = 11 (20/26), n = 7 (20/27) and n = 1 (20/33)]
Patients with NPARMs, n	7
**Clinical data**	
Neonatal versus late-onset CCHS, n	32 versus 2 (diagnosed at 18 months and 9 years)
Isolated CCHS versus CCHS and HD, n	27 versus 7
Night ventilation versus No ventilation, n	33 versus 1
Mask versus Tracheostomy ventilation, n	16 (14 never tracheotomised) versus 17
Phrenic nerve or cardiac pacemaker, n	0
Neonatal convulsions, n	3
Special education program, n	4

Panel B	n of patients tested	Mean ± SD	95% confidence interval
Full-scale IQ	34	82 ± 20	73–91
Verbal comprehension index	33	88 ± 21	79–98
Visual-spatial index	34	88 ± 19	79–96
Fluid reasoning index	33	90 ± 19	82–99
Working memory index	31	81 ± 13	75–87
Processing speed index	31	77 ± 19	70–89

**Fig. 1:**
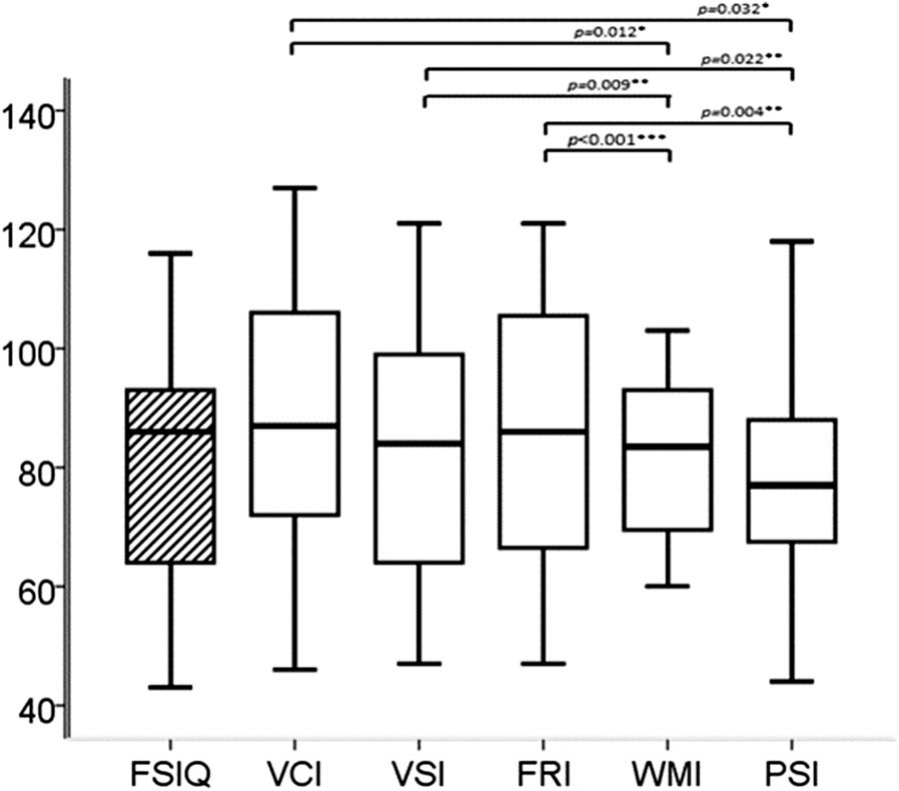
Intellectual measures for the whole group (N = 34). Boxplots show the median in the middle, box lines show the first and third quartiles, and whiskers show the maximum and minimum values. *FSIQ* full-scale intellectual quotient, *VCI* verbal comprehension index, *VSI* visuo-spatial index, *FRI* fluid reasoning index, *WMI* working memory index, *PSI* processing speed index. *p < 0.05; **p < 0.01; ***p < 0.001 (using paired *t* tests)

*Intellectual measures* included Full-scale Intelligence Quotient (FSIQ) and primary indices (Fig. 1). Half of the patients had normal intelligence, but for the whole group, mean (SD) individual FSIQ was 80 (20), being in the low average range (IQ 80–89). Similarly, mean scores of Verbal Comprehension Index (VCI), Visuo-Spatial Index (VSI), Fluid Reasoning Index (FRI) and Working Memory Index (WMI) were in the low average range; only mean score of Processing Speed Index (PSI) of 77 (19) was in the borderline range (IQ 69–79). Of importance, scores of WMI and PSI were significantly lower than those of VCI, VSI and FRI (*p* < 0.001 FRI vs WMI, *p* = 0.004 FRI vs PSI*, p* = 0.009 VSI vs WMI, and *p* < 0.05 for the others, Fig. 1).

### Association between intellectual indices and CCHS characteristics

For the whole group, there was no association between intellectual indices and various CCHS characteristics, e.g. age group, gender, disease onset, presence of Hirschsprung disease.

**Fig. 2:**
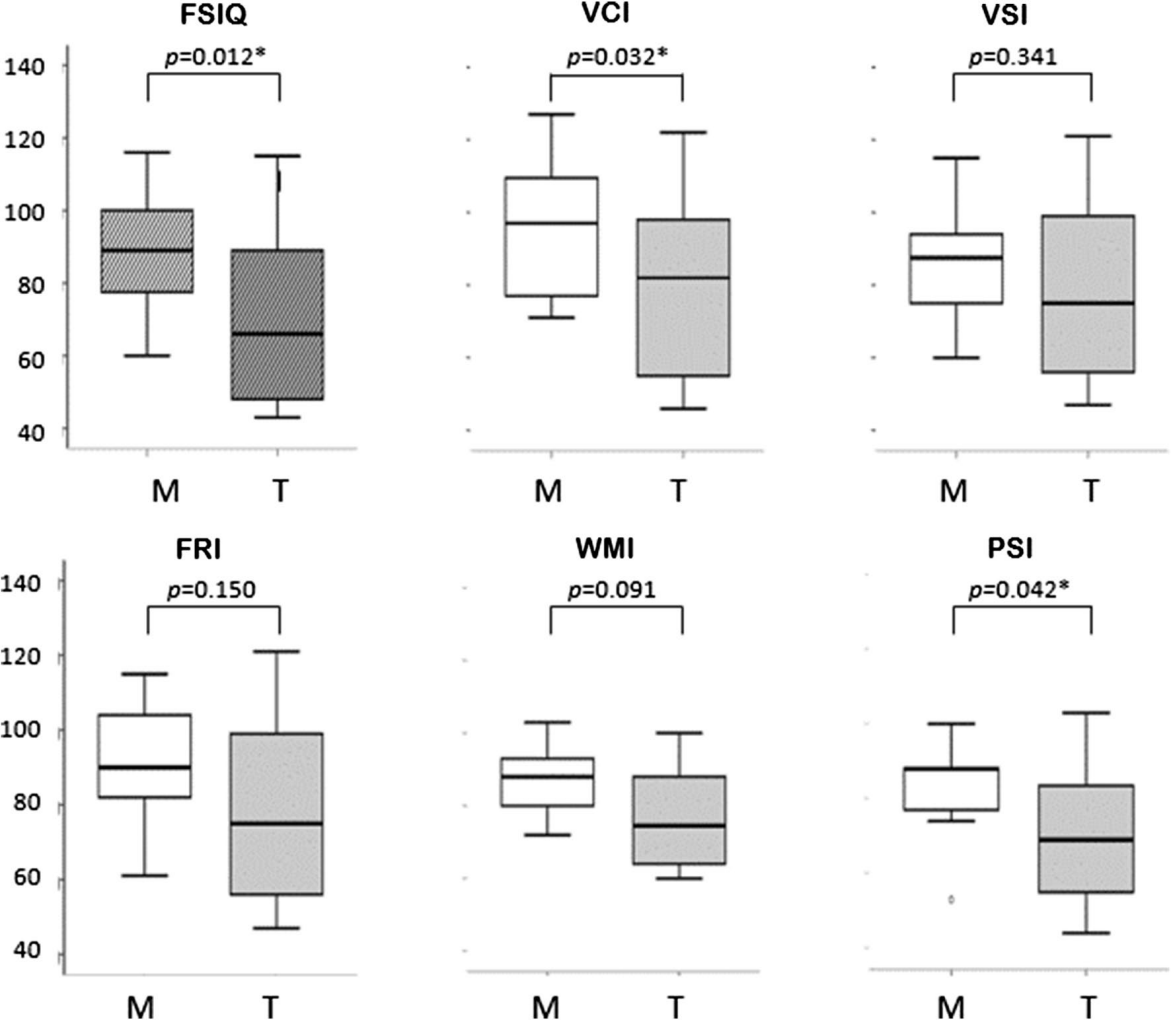
Comparison of intellectual measures between CCHS patients with Mask ventilation (n = 16) and those with Tracheostomy ventilation (n = 17). Boxplots show the median value in the middle, box lines show the first and third quartiles, and whiskers show maximum and minimum values. *M* mask ventilation, *T* tracheostomy ventilation, *FSIQ* full-scale intellectual quotient, *VCI* verbal comprehension index, *VSI* visuo-spatial index, *FRI* fluid reasoning index, *WMI* working memory index, *PSI* processing speed index. *p < 0.05 (using ANOVA tests)

Interestingly, mean scores of intellectual indices were found greater in patients with mask ventilation than in those with tracheostomy ventilation; a level of significance was obtained for FSIQ, VCI and PSI (*p* = 0.012, 0.032 and 0.042 respectively, Fig. 2).

**Fig. 3:**
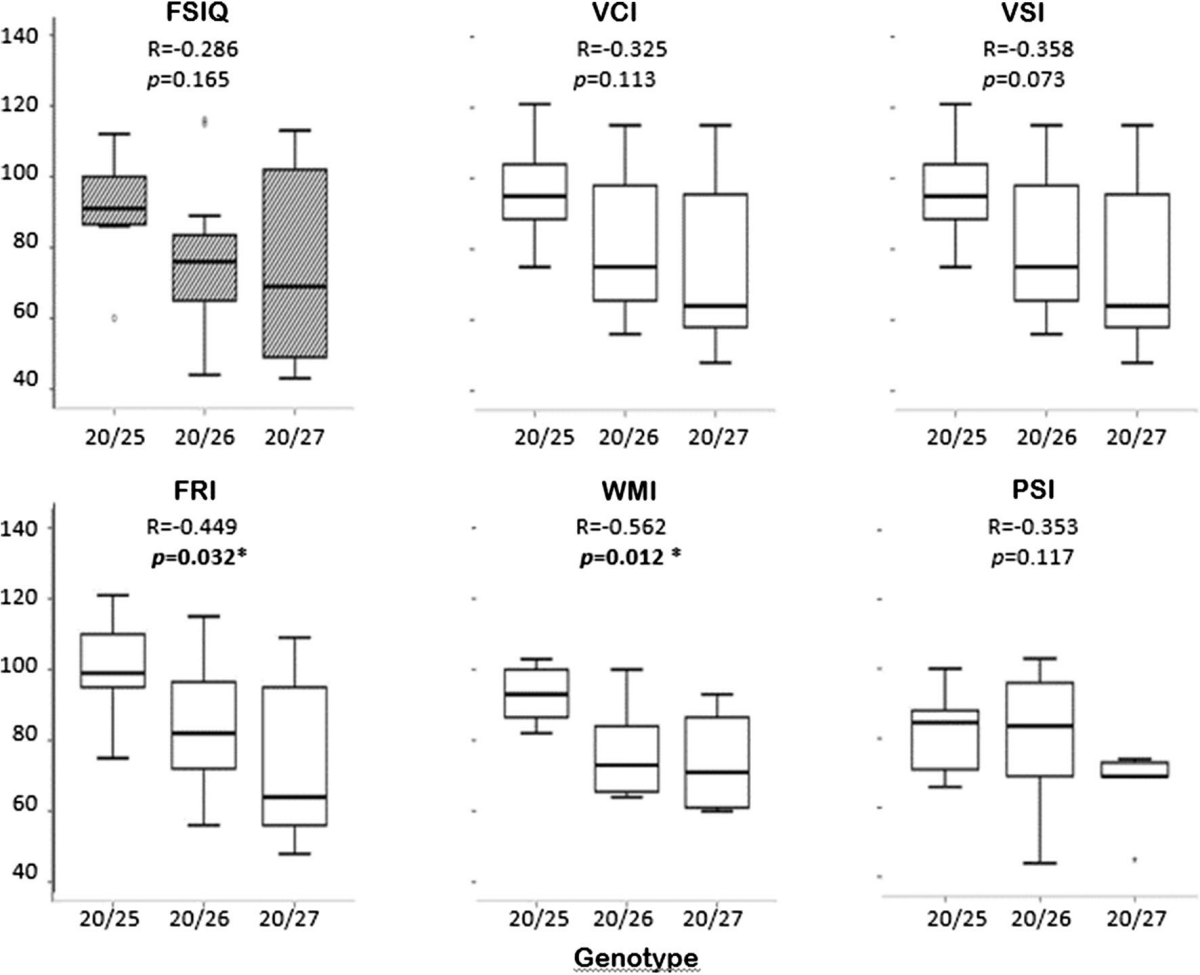
Comparison of intellectual measures and genotypes in the PARM group. Genotypes were 20/25 (n = 8), 20/26 (n = 11) and 20/27 (n = 7). Data of one patient with 20/33 were not shown. Boxplots show the median in the middle, box lines show the first and third quartiles, and whiskers show maximum and minimum values. *FSIQ* full-scale intellectual quotient, *VCI* verbal comprehension index, *VSI* visuo-spatial index, *FRI* fluid reasoning index, *WMI* working memory index, *PSI* processing speed index. *p < 0.05 (Pearson’s correlation tests)

With regard to the genetic status, there was no significant differences in intellectual indices between PARM and NPARM groups and between NPARM, 20/25, 20/26 and 20/27 groups. However, when considering the PARM subgroup only, there was a statistically significant difference between subgroups for WMI as demonstrated by one-way ANOVA (*F*(2,23) = 4.9, *p* = 0.022). A Tukey post hoc test showed that WMI was statistically greater in the 20/25 group than in the 20/26 group (*p* = 0.037) and in the 20/27 group (*p* = 0.045). Most interestingly, there was a negative correlation between each of the intellectual indices and the number of polyalanine expansion. Correlation reached statistical significance for FRI and WMI (*r* = − 0.449, *p* = 0.032 and *r* = − 0.562, *p* = 0.012 respectively, Fig. 3).

## Discussion

The present study showed that patients with CCHS had a mean Full-scale IQ in the low average range and that indexes of both working memory and processing speed were significantly lower when compared to the other Wechsler primary indices. These results were consistent with a few previous publications [17, 24, 30]. Of interest, there were two new findings. Full-scale IQ, and indexes of verbal comprehension and processing speed were found significantly greater in patients with mask ventilation than in those with tracheostomy ventilation. More importantly, in the patients with PARMs, intellectual indices showed negative correlations with the number of polyalanine expansion, with statistical significance reached for indexes of working memory and fluid reasoning. These data may suggest that both genetics and ventilation method may influence neurocognitive impairment in CCHS (Table 2).

**Table 2:** Comparison of intellectual indices between subgroups of CCHS patients using Mask ventilation versus Tracheostomy ventilation

	Mask (n = 16)	Trach (n = 17)	Mean difference	95% CI of the difference	*p*
Age (years)	8.2 ± 3.7 (n = 16)	9.5 ± 3.4 (n = 17)	− 1.2	− 3.8; − 0.7	0.053
FSIQ	89 ± 17 (n = 16)	70 ± 23 (n = 17)	19	4; 34	0.012*
VCI	95 ± 18 (n = 15)	78 ± 24 (n = 17)	17	2; 33	0.032*
VSI	86 ± 17 (n = 16)	79 ± 24 (n = 17)	7	− 8; 22	0.341
FRI	91 ± 15 (n = 13)	79 ± 25 (n = 17)	11	− 4; 27	0.150
WMI	86 ± 10 (n = 14)	77 ± 15 (n = 16)	9	− 2; 20	0.094
PSI	84 ± 14 (n = 15)	70 ± 17 (n = 16)	14	0.5; 27	0.042*

Data are mean ± SD; n is the number of patients tested

*FSIQ* full-scale intellectual quotient, *VCI* verbal comprehension index, *VSI* visuo-spatial index, *FRI* fluid reasoning index, *WMI* working memory index, *PSI* processing speed index

*p < 0.05 (using ANOVA tests)

These results should be understood in the following context. (1) Our study group was the largest reported thus far, investigated using the same standardized battery of cognitive tests. However, knowing the heterogeneity in clinical presentations in CCHS, the sample could still be considered as rather limited. (2) Our study group was the first reported with both patients using invasive ventilation and those using non-invasive ventilation. Invasive tracheostomy ventilation remained preferred for patients with CCHS in many parts of the world. Meanwhile, non-invasive ventilation has widely spread in Europe, mainly in some countries such as France, United Kingdom, Italy or Sweden. In these countries, during the last decade, mask ventilation has been initiated in patients with CCHS even during the neonatal period, so that some of them were never tracheotomised. (3) Although our study group included patients with CCHS, there were some recruitment biases. Health services for CCHS is organised as following in France: a national protocol of diagnosis and management of CCHS serves as guidelines [27], patients are managed by physicians near their place of residence and regularly assessed at the national centre of reference in Paris. This could explain the high proportion of patients with NPARMs in our study group (20% instead of the less than 10% in published cohorts [28]). However, it was unlikely that this had impacted our overall results as there were no significant differences in intellectual measures between PARM and NPARM groups. (4) There was no matched comparison control group and cognitive assessments were not blind to diagnosis, although the neuropsychologist was blind to genetic data at the time of investigation.

Most of our patients with CCHS attended normal school, but many had repeated a school grade or required special education programs. Our results showed that half of them had normal intelligence but a broad variability of intellectual test results was observed. Interestingly, we found that both processing speed and working memory were significantly more affected than the other domains of cognition. Similar findings have been previously reported in school-age children with CCHS [30]. Processing speed refers to the speed with which one can complete simple cognitive tasks automatically and accurately as possible and move to the following one. Working memory is a master cognitive function which temporarily maintains focus on new incoming information before it is processed by the central executive. Working memory is often associated with attention and executive functioning capacities [2]. Research has long showed relationships between processing speed and working memory capacities: indeed, individuals who can process information rapidly do not have to maintain it long in the working memory; conversely, those who process slowly will overload the working memory which can store a limited amount of information for short periods of time only. Different models have been proposed to shed light on the nature of complex interactions between processing speed and working memory and between these with other dimensions such as attention [5, 10]. In all cases, limitations in processing speed and working memory resulted in significant delays in learning processes such as reading, mathematics and expression.

Causes underlying neurocognitive impairment in CCHS have been hypothesized but remained unfully investigated. Decades of research have shown the impacts of various sensory nervous system dysfunctions in learning difficulties. These approaches which are of high clinical relevance in many conditions may prevail in CCHS also. Indeed, the slowness observed in patients with CCHS has been suggested to be caused by visuo-spatial disorders. Patients with CCHS poorly performed visual-motor integration test, only two out of 11 of them were able to score at greater than the 50th percentile of age-matched subjects [24]. Ocular disorders are a prominent feature of CCHS, found in as many as 90% of the patients. They mainly include pupillary defects and accommodative insufficiency as a result of ocular dysautonomia [18]. Most of the patients require corrective glasses or lenses and orthoptic rehabilitation. Whether there is additional dysfunction of the central visual pathways and of related brainstem nuclei has not been investigated yet. Moreover, an amount of literature has shown that reduced processing speed and working memory were associated with language disabilities [10]. In the neonatal period, patients with CCHS often have poor oral sphere motor capacities that affect ability to swallow, to eat then to speak. Vocabulary is developed through social interactions and experiencing new situations. Communication problems often result in deficits in vocabulary skills. Interestingly, the present study showed that patients with tracheostomy had poorer verbal comprehension and lower processing speed than those with mask ventilation. Indeed, tracheostomy ventilation is the only secure mode of ventilation used in CCHS patients with severe clinical presentations and associated co-morbidities. The latter are likely confounding factors leading to poor neurocognition. But having a tracheostomy may be an additional risk factor for greater speech and language delays despite specific therapies and further reduced communication options [25]. In this context, our results may suggest that mask ventilation should be preferred whenever possible in terms of safety, because the integrity of the oral-tracheal sphere may favour the subsequent development of speech and language.

Research has also shown that recurrent hypoxia and/or hypercapnia can play a deleterious role in brain development, mainly in the first years of life, as previously reported in prematurity or congenital heart defects [6, 19]. But relationships between neurocognition and severity of respiratory deficits have not been demonstrated in CCHS [30]. Knowing the role of central autonomic networks on brain functioning, one may wonder in which extent CCHS-related autonomic dysfunction may impact on the modulation of brain development and plasticity, especially during critical periods of childhood. Of great interest, our present study is the first to show negative correlations between intellectual measures and the number of polyalanine expansions, with significance obtained for indexes of working memory and fluid reasoning. Before definitive conclusions can be sought, these results need to be confirmed in larger samples of patients with CCHS. Brain imaging have shown that some brain structures known as involved in neurocognition such as the caudate nucleus, the hippocampus or the ventral medial prefrontal cortex were smaller in CCHS subjects than in the controls [11, 12]. Moreover, although overall cerebral blood flow was increased in CCHS, there were large regional variations with low blood flow especially in areas involved in autonomic regulation. This may reflect vascular alterations or impaired autonomic regulation in these areas [7, 8]. Therefore, one may speculate on putative relationships between central autonomic dysfunction, brain plasticity and neurocognitive deficiencies in CCHS.

## Conclusion

Although rare patients may have remarkable achievements, our findings confirm the importance of neurocognitive symptoms in the neonatal and childhood CCHS. They further suggested that both genetic and ventilation method were involved in the physiopathology of the impaired neurocognition. Further investigations are required to explore and better understand the insights of complex physiopathological processes. Learning difficulties in children with CCHS could be explained by the multiplicity and importance of cognitive deficits, even in those with normal intelligence. They significantly impacted on academic course and social interaction. Assessments of neurocognitive capacities should be performed at early ages and repeated regularly in order to detect subtle deficits and tailor re-educative programs. Remedial plans for improvement of various cognitive dimensions should be investigated. Children with CCHS require close monitoring of ventilatory status and general health and deserve more specialized adaptation of learning processes. Personalized medicine is likely the key concept to ensure academic and social achievements for the patients with CCHS.

## Data Availability

Availability of data and materials is on request.

## Abbreviations

CCHS: Congenital Central Hypoventilation Syndrome
CI: Confidence interval
FRI: Fluid reasoning index
FSIQ: Full-scale IQ
HD: Hirschsprung disease
IQ: Intelligence quotient
NPARM: Non polyalanine repeat mutation
PARM: PolyAlanine repeat mutation
PHOX-2B: Paired like homeobox 2b gene
PSI: Processing speed index
VCI: Verbal comprehension index
VSI: Visuo-spatial index
WISC: Wechsler Intelligence Scale for Children
WMI: Working memory index
WPPSI: Wechsler for Preschool and Primary Scale for Intelligence
